# Gradient Nanostructured Tantalum by Thermal-Mechanical Ultrasonic Impact Energy

**DOI:** 10.3390/ma11030452

**Published:** 2018-03-20

**Authors:** Jong-Min Chae, Keun-Oh Lee, Auezhan Amanov

**Affiliations:** 1 Department of Safety Engineering, Seoul National University of Science and Technology, Seoul 01811, Korea; jmchae72@daum.net (J.-M.C.); leeko@seoultech.ac.kr (K.-O.L.); 2 Department of Mechanical Engineering, Sun Moon University, Asan 31460, Korea

**Keywords:** tantalum, hardness, gradient nanostructured layer, grain size, residual stress, dry wear behavior

## Abstract

Microstructural evolution and wear performance of Tantalum (Ta) treated by ultrasonic nanocrystalline surface modification (UNSM) at 25 and 1000 °C were reported. The UNSM treatment modified a surface along with subsurface layer with a thickness in the range of 20 to 150 µm, which depends on the UNSM treatment temperature, via the surface severe plastic deformation (S^2^PD) method. The cross-sectional microstructure of the specimens was observed by electron backscattered diffraction (EBSD) in order to confirm the microstructural alteration in terms of effective depth and refined grain size. The surface hardness measurement results, including depth profile, revealed that the hardness of the UNSM-treated specimens at both temperatures was increased in comparison with those of the untreated ones. The increase in UNSM treatment temperature led to a further increase in hardness. Moreover, both the UNSM-treated specimens with an increased hardness resulted in a higher resistance to wear in comparison with those of the untreated ones under dry conditions. The increase in hardness and induced compressive residual stress that depend on the formation of severe plastically deformed layer with the refined nano-grains are responsible for the enhancement in wear resistance. The findings of this study may be implemented in response to various industries that are related to strength improvement and wear enhancement issues of Ta.

## 1. Introduction

Tantalum (Ta) is a rare, refractable, malleable, and lustrous metal, which is widely used in various industries, in particular, aerospace, electronic devices, and nuclear applications, owing to its high ductility at temperatures below 150 °C, good forging, and chemical and physical properties [1]. Especially, Ta exhibits a superior corrosion resistance due to a natural protective layer created by oxides of Ta on its surface [2]. The chemical inertness of Ta makes it an ideal substance for equipment and a substitute for platinum (Pt) [3]. Ta is a highly bioinert metal to manufacture biomedical components, such as hip, knee joints, and other orthopaedic implants because it is not harmed by bodily fluids and also does not irritate the flesh of the implant. The elasticity of Ta helps to avoid stress shielding of hip and knee replacements as well [4]. Ta is also a candidate material to be used in prostheses instead of Ti in the near future. In spite of the facts that Ta is a highly corrosion resistant, chemically inert, etc., but its main advantages are low strength, low wear resistance, and low fatigue strength, which may prevent its successful potential applications in a wide variety energy and fatigue ranges, starting from biomedical through chemical process equipment. The realization of components made of Ta suited for harsh and high-temperature conditions is a challenge since it is problematic to control the required mechanical properties and the fatigue strength of Ta in aerospace applications, such as gas turbines or engines where the temperature rises up to 650 °C [5]. In general, a usage of Pt is a possible option due to its high chemical inertness and high temperature stability, where Ta can be substituted for more expensive Pt, but it is not considerable from the economic and commercial point of view. Therefore, an increase in strength and wear performance, and also an extension in service life of Ta are in high demand. 

One of the easy and possible ways to control the wear performance of metallic materials by increasing its strength is controlling its microstructure, in particular, producing nano-grains with grain boundaries of mostly high angle misorientation via surface severe plastic deformation (S^2^PD), which is a cold-forging process [6,7]. A wide range of S^2^PD processes, such as shot peening (SP) [8], surface mechanical attrition treatment (SMAT) [9], surface rolling treatment (SRT) [10], and ultrasonic shot peening (USP) [11], and severe plastic deformation (SPD) processes, such as equal channel angular processing (ECAP) [12] and high-pressure torsion (HPT) [13] were developed in the past. These processes develop a severe deformation and high strain, which cause the creation of gradient micro- and sub-micrometer grains, whose size is gradually increasing with the depth and finally reaches the actual initial size of matrix and coarse grains in a certain of depth [10,13]. As a consequence, sufficient S^2^PD and SPD processes result in the apparent modification in microstructure in terms of highly misoriented nano-sized grains. The mechanism behind the nanocrystallization and grain refinement via S^2^PD and SPD processes lies shear bands associated with the dynamic recrystallization (DRX) [14]. The results of S^2^PD and SPD processes, such as microstructural state alteration, are strongly responsible for the subsequent increase in mechanical properties of metallic materials, which in turn has a direct correlation to the wear performance of metallic materials [6]. S^2^PD and SPD processes are capable of increasing the hardness, yield strength, and elastic strain featuring tendency to saturation, but it is not beneficial in terms of ductility [15]. Generally, nano-sized grains have some benefits in comparison with the coarse grains, not only in terms of strength of a material, but also superplastic deep drawing. 

A wide range of metallic materials such as aluminum [6], titanium [7,9,12], stainless steel [8], Inconel [11], etc. were subjected to S^2^PD and SPD processes to achieve a refined nano-grained and nanocrystalline structure in the past, but only a few limited studies concerning the application of those processes to Ta can be found in the literature. For example, Huang et al. studied the effects of HPT on microstructure and the hardness of pure Ta [16]. It was found that the grain size was refined significantly due to the increase in hardness, but some coarse grains appeared with increasing the numbers of rotations. Mathaudhu et al. produced a fine grained Ta via multi-pass equal channel angular extrusion (ECAE) process [17]. It was concluded that the refined nano-grains with the size of 100–400 nm was found to be beneficial for Nb_3_Sn superconductor. Another study by Mathaudhu et al. on grain size refinement of Ta by ECAE process has attracted wide attention in view of a variety of findings [18]. It was found that ECAE process refined grains at *ε* = 2.3, which is more important than initial grain size to occur a recrystallization. Moreover, Zhang et al. reported the possibility of generating a nanostructured Ta with a grain size of 20 nm via sliding friction treatment (SFT) [19]. Nevertheless, there are no systematic investigations on the mechanical properties and wear performance of Ta treated by ultrasonic nanocrystalline surface modification (UNSM) technique [20]. A precision control of UNSM technique is a key advantage to produce a gradient nanostructured material over other SPD and S^2^PD processes. The UNSM treatment used to be applied for various metallic materials, ceramics, Si wafer, various coatings to improve the friction behavior, wear and corrosion resistance, and fatigue strength through the presence of nanostructured surface layer with the refined nano-sized grains [21,22,23,24,25]. Furthermore, a high-temperature UNSM treatment along with local heat treatment (LHT) was developed and recently patented, where the UNSM treatment temperature can be reached up to 1400 °C. Interestingly, so far it was discovered that the hardness and wear performance of Ti-6Al-4V alloy was improved via UNSM treatment at a high temperature [26]. However, the hardness can be continuously increased with temperature, where a softening may occur as well. For example, a softening occurred in Ni-based superalloy (Inconel 690 alloy) when the high-temperature UNSM treatment was performed at 700 °C [27]. Moreover, the highest hardness of α-Ta treated by high-temperature UNSM treatment was found at 800 °C [20]. It is therefore of interest to further increase the high-temperature UNSM treatment temperature up to 1000 °C, and to investigate its effects on the grain size refinement, hardness, and wear resistance of Ta since the increase or decrease in hardness and also grain size refinement or grain growth depend on the nature of a material. In this regard, the main purpose of the investigation is to provide systematic experimental results on the grain size refinement, hardness, and wear performance of Ta that is treated by UNSM treatment at room temperature (RT) and high temperature (HT) of 1000 °C. It is strongly believed, according to the results, that the role of a high-temperature UNSM in Ta-related applications, such as aerospace, nuclear, electrical, etc., will be significantly important. 

## 2. Materials and Methods 

### 2.1. Specimen Preparation 

Refractory Ta is rarely used since alloying makes other metals brittle, with an exception of steel, in which case it increases the ductility and strength. In this study, the specimens with dimensions of 20 × 5 mm^2^ was prepared from bulk Ta with a hardness of 195 ± 6 HV (after cold working). Important properties of Ta are shown in Table 1. 

### 2.2. Ultrasonic Nanocrystal Surface Modification (UNSM) and Local Heat Treatment (LHT)

The UNSM parameters that are shown in Table 2 were selected to treat the specimens at RT and HT (1000 °C) temperatures. At HT, the specimens were heated up with a halogen-based high-temperature heating system, where the actual temperature was measured using a portable pyrometer. More detailed information about the UNSM technique, including high-temperature heating setting of LHT, can be found in our previous publications [26,27]. Following the UNSM treatment, both of the disk specimens were mounted in bakelite and were polished with sand papers down to 2400 grit, and then a colloidal solution was used with a powder of 1 µm in diameter to achieve a mirror-like surface. Afterwards, the disk specimens were electrolytically etched in H_2_O, H_2_SO_4_, HF with a couple of drops of H_2_O_2_ solution at 5 V for 30 s using an electropolisher-etcher (ElectroMet^TM^4, Buehler, Uzwil, Switzerland) to reveal the microstructural features, such as grains, grain boundaries, etc. 

### 2.3. Wear Behavior in Dry Conditions

A commercially available ball-on-disk tribometer (Anton Paar, Graz, Austria) was used to evaluate the wear performance of Ta that came into contact with a steel ball under the test conditions, as shown in Table 3. Each test was replicated three times. The surface roughness (*R_a_* < 0.08 µm) of the specimens was considered to be nearly close to escape from the influence of surface roughness on the wear performance under dry conditions. 

### 2.4. Characterizations

The surface hardness of the specimens was measured using a micro-Vickers hardness tester (MVK E3, Mitutoyo, Takatsui, Japan) at a load of 300 gf for dwell time of 10 s, while the nanoindentation was performed using a depth sensing tester (MTS, Nanoindenter XP, Eden Prairie, MN, USA), with a diamond Berkovich indenter at a frequency of 45 Hz, strain rate of 0.05 s^−1^ with a maximum load of 100 mN. The average surface roughness *(R_a_)* and cross-sectional wear track profiles to quantify the wear rate were measured using a portable two-dimensional surface profilometer (SJ-210, Mitutoyo, Japan). X-ray diffraction (XRD) was performed with a CuKα radiation (*k* = 1.5418 Å) at a wavelength of 1.54, a tube current of 40 mA, and a voltage of 30 kV using a sin^2^ψ method over the range of 20–130°, with a scanning speed of 1°/min using a Bruker D8 Advance X-ray diffractometer to measure the residual stress and to identify phases before and after UNSM treatment. The dimension of the specimen was 10 × 10 × 3 mm^3^. The residual stress was also measured by using an indentation method that was based on the nanoindentation results. The obtained residual stress measurement data with a huge error bar represent the average of three measurements. A gradient nanostructured surface layer was observed using an electron backscatter diffraction (EBSD: Oxford Instruments HKL Nordlys Max, Abingdon, UK) installed into a scanning electron microscopy (SEM: JEOL 6610LV, Tokyo, Japan) at an accelerating voltage of 20 kV with a large beam current of 10 nA. Surface microstructure and wear tracks, and the chemical composition were characterized by SEM, along with energy-dispersive X-ray spectroscopy (EDX: EDAX/AMETEK, Mahwah, NJ, USA). 

## 3. Results and Discussion

### 3.1. Microstructural Evolution by EBSD 

**Figure 1:**
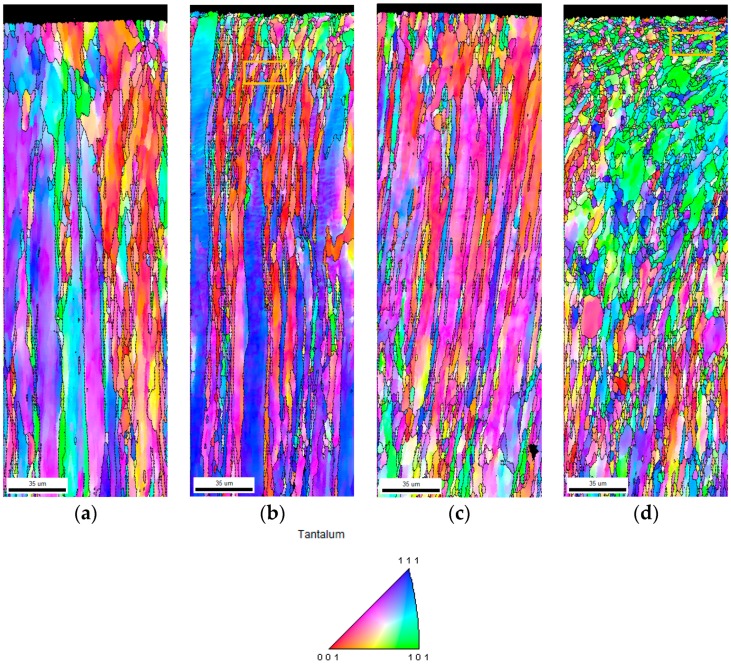
Cross-sectional electron backscatter diffraction (EBSD) Inverse Pole Figure (IPF) maps of the untreated (**a**), UNSM-treated at RT (**b**) and local heat treatment (LHT) without (**c**) and with (**d**) UNSM treatment at 1000 °C specimens.

Cross-sectional Inverse Pole Figure (IPF) maps of the specimens obtained using an electron backscattered diffraction (EBSD) are shown Figure 1. The untreated specimen, as shown in Figure 1a, presents a homogenous microstructure with equiaxed grains at the topmost surface in the range of 10–30 µm in diameter. Obviously, it was observed that the UNSM treatment at RT generated a gradient nanostructured surface layer, as shown in Figure 1b. In addition, an effective depth of UNSM treatment at RT was found to be about 35–40 µm, while some refined grains are also randomly visible in a depth of about 100 µm between the elongated coarse grains with further increasing the depth beyond the actual effective depth of UNSM treatment at RT. This irregular deformation may be attributed to the inhomogeneous of a large amount of plastic deformation that was introduced during UNSM treatment. Figure 1c shows the IPF color map of the specimen LHT at 1000 °C without UNSM treatment. It can be seen that some elongated coarse grains at the top surface were refined remarkably, which may be a result of machining and mechanical polishing. Figure 1d shows the IPF color map of the specimen LHT at 1000 °C with UNSM treatment. The modification in microstructure with depth can be further extended by performing an LHT with UNSM treatment at 1000 °C in comparison with the UNSM-treated specimen at RT. The effective depth was found to be deeper than ~150 µm, which is about five times deeper than that of the UNSM at RT specimen. Interestingly, elongated coarse grained was refined due to the results of heat treatment, but both grain size refinement and extension in effective depth occurred by performing an LHT with UNSM treatment at 1000 °C (see Figure 1c). Moreover, it was observed that a lamellar structure with low-angle grain boundaries (LAGB) is visible (see Figure 1b,d)). It is worth mentioning here that no banded structures, such as deformation and shear, were observed in the untreated and LHT at 1000 °C without UNSM treatment specimens. Microbands are visible feature in the both UNSM-treated at RT and HT specimens, as shown by the dashed lines in Figure 1b,d. These banded structure produced by UNSM treatment is an important factor in high stacking fault energy (SFE) metallic materials subjected to deformation [28]. In general, α-Ta has needle-shape morphology, while β-Ta has a spherical-shaped one [1]. As shown in Figure 1, the elongated shape of needle-shape grains of α-Ta was changed into the spherical-shaped grains, owing to ultrasonic-based strikes at a frequency of 20 kHz. 

**Figure 2:**
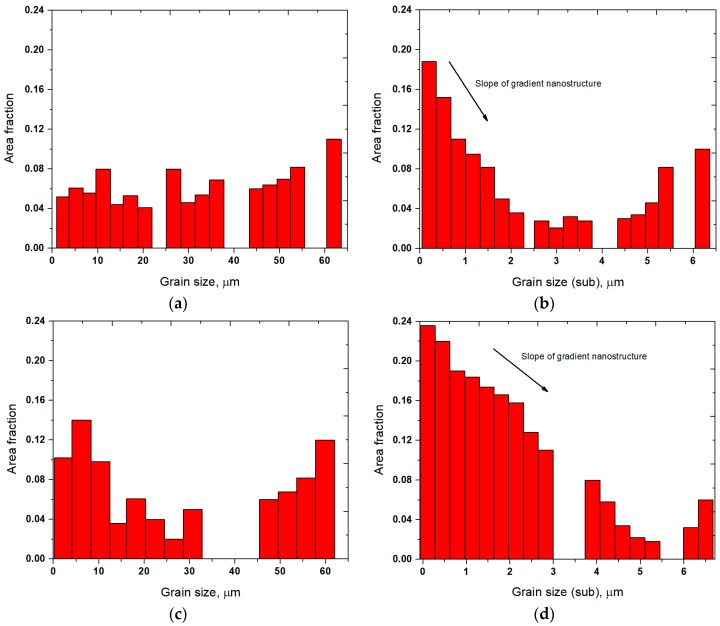
Histogram of the grain size distribution of the untreated (**a**), UNSM-treated at RT (**b**) and LHT without (**c**) and with (**d**) UNSM treatment at 1000 °C specimens representing the presence of gradient nanostructured surface layer.

The grain size distributions with area fraction of the specimens are presented in Figure 2. The grain size distribution of the untreated specimen (see Figure 2a) shows that the grains in the range of 5–60 µm are distributed uniformly, while the UNSM treatment at RT was capable of producing a gradient nanostructure layer with a high fraction (~18.8%) of (sub) grains with a size less than 0.1 µm at the topmost surface, which is deliberately increased, as shown in Figure 2b. The fraction of grains of the specimen treated by solely LHT treatment (without UNSM treatment) was found to be higher in comparison with the untreated specimen, as presented in Figure 2c. The grain fraction was increased significantly by high-temperature UNSM treatment at 1000 °C, as shown in Figure 2d and also the slope (angle) of a gradient nanostructure was higher in comparison with the UNSM-treated specimen at RT. Gradient nanostructured materials have a number of advantages over the homogeneous nanostructured materials in terms of mechanical properties, especially ductility. For example, Lu has discovered the possibility of making a balance between the strength and ductility of materials by producing a gradient nanostructured material [29]. Kang et al. have also demonstrated the features and importance of a gradient nanostructured material that is produced by high pressure torsion (HPT) [13]. It was concluded that the gradient nanostructured material had a significant higher strength with no loss in ductility in comparison with the nanostructured material. Yin et al. have also produced a gradient nanostructure surface layer on Cu by SMAT, pointing out that a gradient nanostructure can exhibit superior strength-ductility synergy [30]. Recently, Wang et al. reported the possibility of generating a gradient nanostructured surface layer in Cu with a grain size of 85 nm via rotationally accelerated shot peening (RASP) [31]. This newly developed RASP technology may apply much higher impact energy in comparison with the conventional SP process. Yang et al. investigated the role of volume fraction of gradient nanostructures Cu that is produced by SMAT at cryogenic temperature [32]. It was found that the gradient nanostructure exhibited a great correlation between strength and ductility. Wu et al. combined gradient nanostructure with transformation-induced plasticity that is produced by SMAT to synthesized gradient nanostructure in austenitic 304 stainless steel [33]. As a result, a gradient nanostructured layer provided a good correlation between strength and ductility. Therefore, it is now desirable to produce a gradient nanostructured material with a thick layer as much as possible as the homogeneous nanostructured material due to the lack of ductility. Moreover, AlMangour and Yang improved the mechanical properties of 17-4 steel that was fabricated by direct metal laser sintering (DMLS), which is a type of additive manufacturing (AM), through grain size refinement by means of SP [34]. It was reported that severe plastically deformed layer along with grain size refinement by SP led to an increase in mechanical properties. 

### 3.2. Residual Stress and XRD Pattern

**Figure 3:**
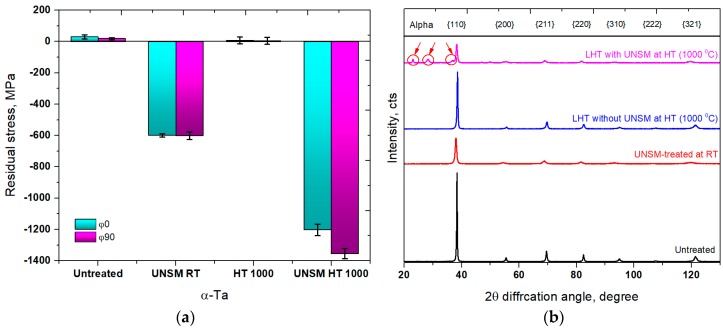
Variation in surface residual stress (**a**) and X-ray diffraction (XRD) pattern (**b**) of the untreated, UNSM-treated at RT and LHT without and with UNSM treatment at 1000 °C specimens.

A comparison in residual stress of the specimens measured at φ0° and φ90° is presented in Figure 3a. It is apparent that the untreated and heat up without UNSM treatment specimens exhibited a tensile residual stress, while the UNSM treatment at both RT and HT induced a great compressive residual stress. The value of the compressive residual stress of the UNSM-treated at RT specimens that were measured both perpendicular and along orthogonal directions was about −600 MPa, which was reached a greater −1200 and −1375 MPa by using an LHT with UNSM treatment at 1000 °C, respectively. Consequently, the compressive residual stress of both the UNSM-treated at RT and HT specimens that were measured along orthogonal direction of φ90° had a higher compressive residual stress in comparison with the compressive residual stress measured along orthogonal direction of φ0°. This difference is due to the different number of strikes. It is well established that the induced compressive residual stress is the most crucial property, which determines the strength and fatigue lifespan of a material [35,36]. It was also reported earlier that SP and USP processes induces high compressive residual stress in the surface layer thanks to the severe plastically deformed layer [11,34]. 

XRD patterns of the specimens are presented in Figure 3b. The relative intensities of all the diffraction peaks of the untreated specimen were reduced significantly after UNSM treatment at RT. By treating the specimen by LHT without UNSM treatment at HT led to a negligible reduction in intensity in comparison with the untreated specimen, but a substantial reduction in intensity was found for the specimen by LHT with UNSM treatment at HT, which is lower in comparison with the UNSM-treated specimen at RT. Post-polishing process of the specimens treated by LHT with and without UNSM treatment at HT was responsible for the absence of any diffraction peaks of the oxide particles on the surface. The high-temperature UNSM treatment, regardless the treatment temperature, may result in the formation of thick oxide layer, which is responsible for the deteriorated mechanical properties [26]. In addition, new diffraction β diffraction peaks {101}, {400} and {410} are detected after LHT with UNSM treatment at HT (see the inset in Figure 3b), which means that the phase transformation occurred from α → β. Beta Ta is a metastable phase that is transformed from α Ta when it heated up to 900 °C [37]. The α phase is tend to have excellent corrosion, thermal ductility properties, while the β Ta provides additional hardenability, therefore the newly appeared β phases are expected to beneficially affect the strength, wear performance, and fatigue strength. 

**Figure 4:**
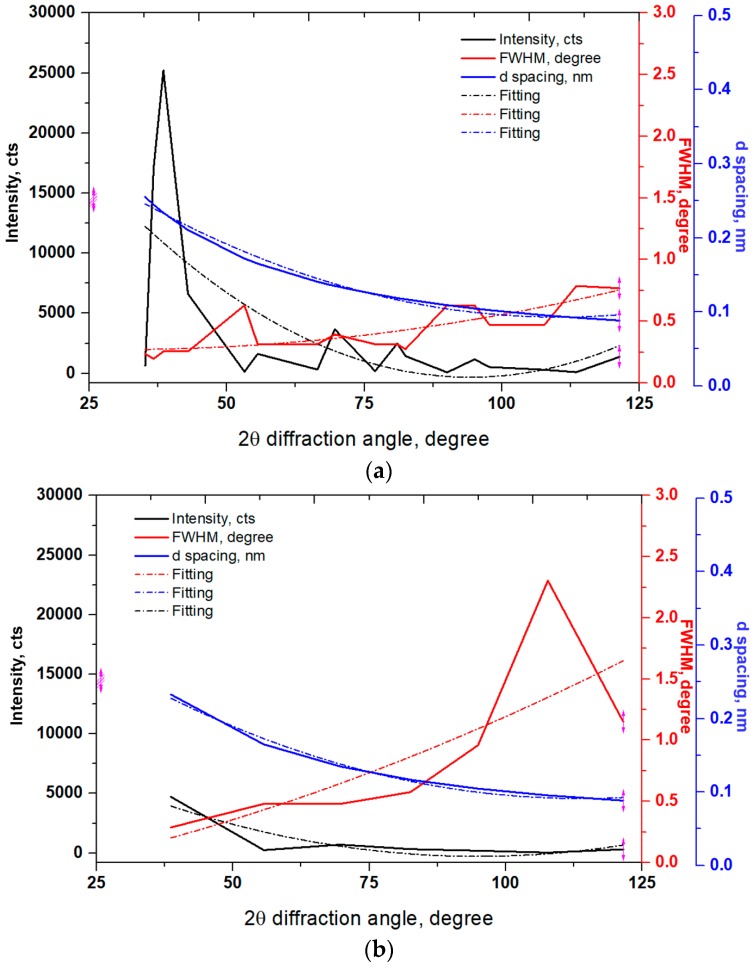

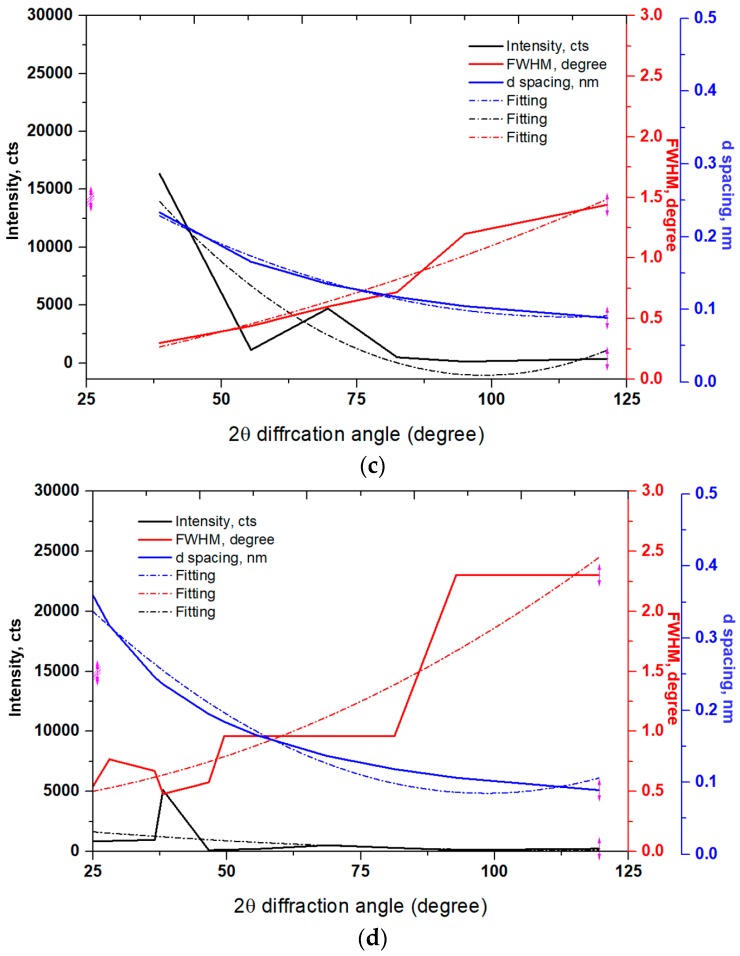
Variation in intensity, full width at half maximum (FWHM) and spacing of the untreated (**a**), UNSM-treated at RT (**b**) and LHT without (**c**) and with (**d**) UNSM treatment at 1000 °C specimens.

A comparison in relative intensity, full width at half maximum (FWHM) and d spacing with respect to diffraction angle of the specimens is presented in Figure 4. It is clear that the relative intensity of the primary alpha peak {110} diffracted at an angle of 38.7 was about 25,000, 5000, 16,700, and 4900 for the UNSM-treated at RT, and LHT with and without UNSM treatment at HT specimens, respectively. Other secondary {200}, {211}, {220}, {310}, {222}, and {321} peaks also reduced remarkably by UNSM treatment both at RT and HT, where they were not visible in Figure 4, while those peaks were reduced as well after LHT without UNSM treatment. In addition, it is noticeable from Figure 4 that the FWHM of the UNSM-treated specimens at both RT and HT got broadening in comparison with the untreated specimen, where the FWHM was increased with increasing the diffraction angle. The highest FWHM of the UNSM-treated specimens at both RT and HT was found to be about 2.5° at a diffraction angle of 108°, as shown in Figure 4b,d. From this, it is clear that the FWHM depends on the relative intensity of the peaks, where the lower relative intensity the higher FWHM. As shown in Figure 4, no significant difference in d spacing of the specimens was found, which is related to the change of diffraction peak position through Bragg’s law [38], where it was gradually reduced with increasing the diffraction angle because when the UNSM treatment applied load is removed, the d spacing returned to normal position unless a compressive residual stress induced by UNSM treatment controls the original strain. It is worth mentioning here that the changes, such as strain, work hardening, etc. of the UNSM treatment at both RT and HT specimens can be estimated by quantitatively analyzing the broadening in FWHM and reduction in relative intensity of diffraction peaks [39]. Top surface grain size of the specimens quantified based on the Scherer equation was in consistent with the cross-sectional EBSD IPF maps, where the refinement of coarse grains into (sub) grains is clearly seen in Figure 1b,d, where the refined (sub) grain size by UNSM treatment at RT was further refined with increasing the temperature, leading to the highest fraction (~23.8%) of sub (grains) at the top surface. Apparently, it is expected to achieve more grain size refinement towards ultrafine grain (UFG) scale with a high-angle grain boundaries (HAGB) by increasing the temperature of UNSM treatment, which can be reached up to 1400 °C so far. In order to confirm the presence of UFG scale at the top surface since the EBSD method does not allow due to the resolution, some advanced quantitative surface analysis by transmission electron microscopy (TEM) is in need to determine the exact nano-sized grain. 

**Figure 5:**
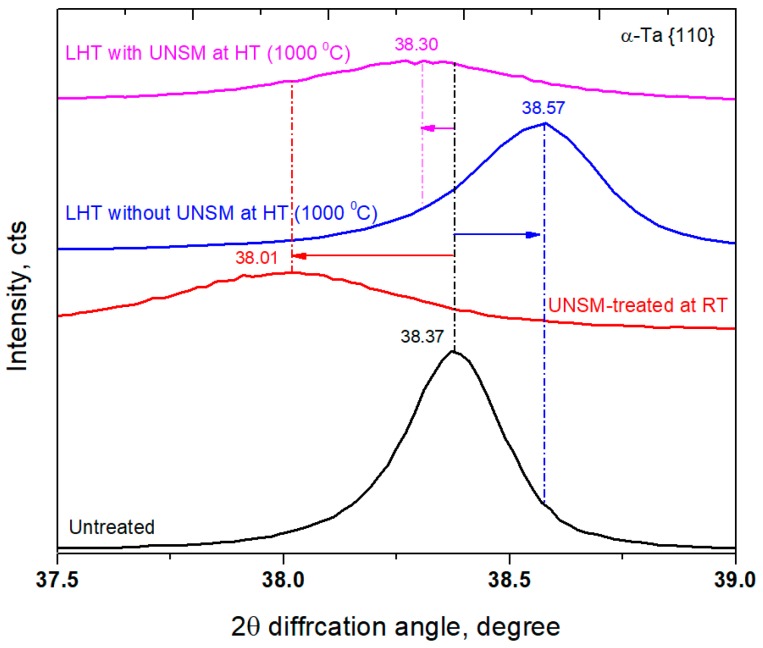
Comparison in intensity peak {110} shift to lower and higher angles of the untreated, UNSM-treated at RT, LHT without and with UNSM treatment specimens.

Furthermore, the diffraction peaks of the UNSM-treated specimens at both RT and HT shifted to a lower diffraction angle (see Figure 5), which is an indicator of the induced compressive residual stress [40], while on the contrary, the diffraction peak of the LHT without UNSM treatment at 1000 °C specimen shifted to a higher diffraction angle. The presence of uniform compressive strain that was derived during grain size refinement process is responsible for the diffraction peak shift to a lower angle [41], while the diffraction peak shift to a higher angle is responsible for the tensile stress [42]. It is well established that the internal stresses, planar faults (stacking faults or twinning) are responsible for the changes in relative diffraction peaks (intensity, FWHM, and shift) of the metallic materials that are subjected to both S^2^PD and SPD processes [43,44]. 

### 3.3. Microhardness and Nanoindentation 

**Figure 6:**
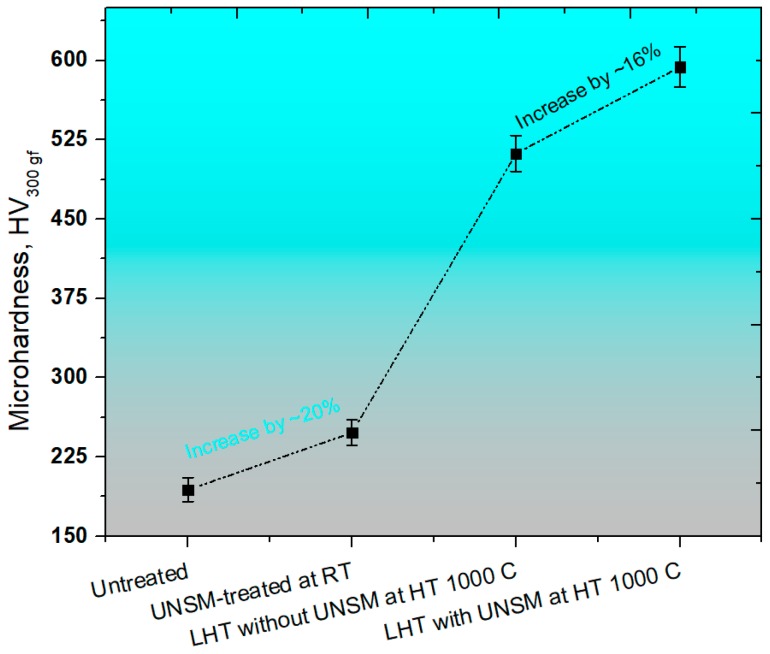
Comparison in surface hardness of the untreated, UNSM-treated at RT and LHT without and with UNSM treatment at 1000 °C specimens.

A comparison in surface hardness of the specimens is shown in Figure 6*.* The UNSM treatment at RT led to an increase in hardness by about 20% in comparison with the untreated one. In turn, the hardness was further increased from 193 to 511 HV by heating up the specimen up to 1000 °C, which is corresponding to a 62% increase in comparison with the untreated one. The untreated specimen that was treated by the combination of an LHT with UNSM treatment was able to further increase the surface hardness by about 16%, 58%, and 70% in comparison with the LHT specimen without UNSM, UNSM-treated at RT, and untreated ones, respectively. The increase in surface hardness by UNSM treatment at both RT and HT is associated with the grain size refinement, which may be explained well by the Hall-Petch relationship, where the grain size is a key factor, in other words, the smallest grain size the highest hardness [45], while the increase in hardness by LHT is related to the movement of atoms from their original position [46]. However, Chokshi et al. have reported a negative slope, where the hardness or strength of a material start dropping with reducing the grain size less than 10 nm due to the grain boundary sliding [47]. Therefore, it is always desirable to refine the grain size bigger than that critical value. 

**Figure 7:**
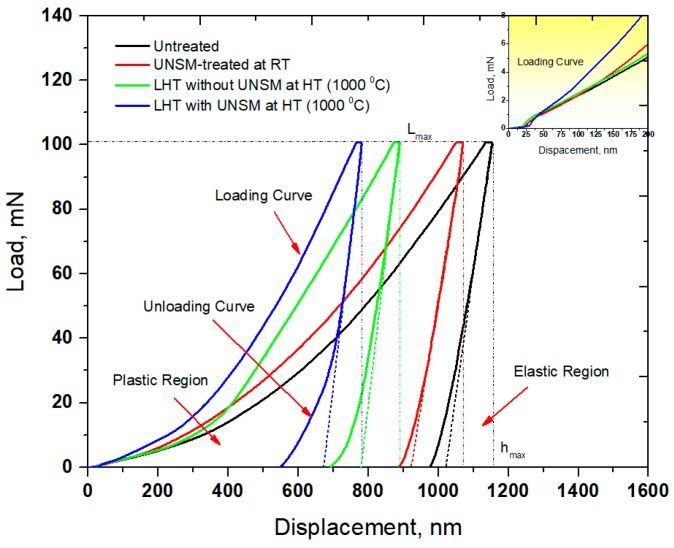
Comparison in load-displacement curve of the untreated, UNSM-treated at RT and LHT without and with UNSM treatment at 1000 °C specimens.

Load and depth of penetration curve of the specimens that was obtained by nanoindentation method is depicted in Figure 7. The UNSM-treated at RT specimen had a shallower depth of penetration in comparison with the untreated specimen under the same load of 100 mN. The depth of penetration occurred on the surface of the LHT without UNSM specimen at HT got shallowed at the same load in comparison with both the untreated and UNSM-treated at RT specimens due to the higher surface hardness, as shown in Figure 6. It was noticed that the UNSM-treated at HT specimen exhibited two times shallower penetration depth in comparison with the untreated one. The increase in hardness by UNSM treatment at both RT and HT thanks to the generation of a gradient nanostructured surface layer and a relatively high dislocation density [13,19,30,48]. Estimation of residual stress is made by various measurement methods, such as XRD, ultrasonic, neutron diffraction, strain curve, magnetic, hole drilling, and Raman spectroscopy as well [49], but these methods have some drawbacks in terms of spatial resolution, data accuracy, and reliability. Zhu et al. discovered a new method to measure the residual stress by the nanoindentation method [50]. The inset in Figure 7 shows the magnified loading curves of the specimen at the onset of the indentation. According to the conclusion of the study [51], the UNSM-treated at RT and LHT with UNSM treatment at HT specimens exhibited a compressive residual stress since both of these specimens required a higher load to be indented in comparison with the untreated and LHT without UNSM treatment at HT specimens, as shown in Figure 7. In turn, the LHT with UNSM treatment at HT specimen required larger load in comparison with the UNSM-treated at RT because of the higher induced compressive residual stress, as shown in Figure 3. In order to validate the results of residual stress that was measured by XRD and nanoindentation methods, a newly proposed nanoindentation method [51,52], which was adopted based on the difference between the contact areas of the specimens, was used using the following equations [51]:

for tensile residual stress
  (1) $$\sigma_{r}=H\left(1-A_{0}/A\right)$$
for compressive residual stress
  (2) $$\sigma_{r}=H\left(1-\frac{A_{0}}{A}\right)/f$$

where *A* and *A*_0_ are the indentation contact area of the specimens with tensile and compressive residual stress (*σ_r_*), respectively. *H* is the material hardness. *f* = sin *α* is a geometric factor, where *α* is related to the indentation angle of the indenter. For a Berkovich indenter, *α* = 24.7° and *f* = 0.418.

**Figure 8:**
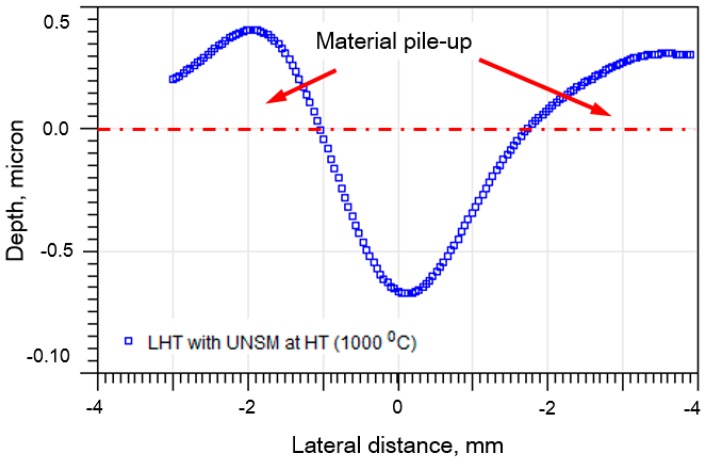
Cross-sectional profile of the residual indent on the surface of the UNSM-treated at RT specimen.

After obtaining a contact area of the specimens with tensile and compressive residual stresses, the tensile residual stress of the untreated and LHT without UNSM treatment at HT, and the UNSM-treated at RT and HT specimens was calculated by Equations (1) and (2), respectively. It was found that the calculated tensile residual stress of the untreated and LHT without UNSM treatment at HT was 24.6 ± 9 and 9.4 ± 3 MPa, while the tensile residual stress that was measured by the XRD method was 20.47 ± 7 and 6.54 ± 2 MPa, respectively. The calculated compressive residual stress of the UNSM-treated at RT and HT specimens was −636.26±86 and −1284.71 ± 114 MPa, while the compressive residual stress that was measured by XRD method was −607.44 ± 82 and −1228 ± 59 MPa, respectively. It is apparent from the residual stress results that were obtained by the nanoindentation method are in good consistence with the residual stress results obtained by XRD method ensuring a standard deviation in the range of about 10–14%. It is worth mentioning here that the residual stress of Au/TiW bilayer was estimated by deflection of double clamped beams, where the beam deflection was corrected by the indent penetration [53]. This method can also be adopted to Ta as well. As a consequence, the residual stress measurement results of the specimens by nanoindentation method are applicable to predict the induced compressive residual stress by UNSM treatment. Additionally, it is important to mention here that this method can be adopted only to materials that create a pile-up around the indent after nanoindentation. The cross-sectional profile of the residual indent on the surface of the UNSM-treated at RT specimen is shown in Figure 8, where the pile up around the indent after nanoindentation is clearly observed. Hence, the calculated tensile and compressive residual stress results by nanoindentation method in this study were found to be absolutely accurate and reliable.

### 3.4. Friction and Wear Performance

**Figure 9:**
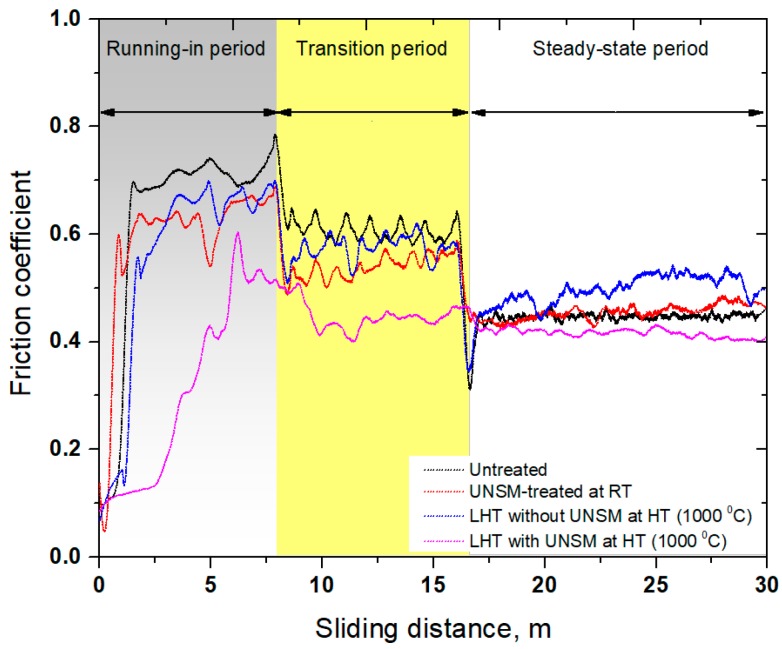
Comparison in friction coefficient with respect to sliding distance of the untreated, UNSM-treated at RT and LHT without and with UNSM treatment at 1000 °C specimens.

Friction of metallic materials is usually relatively high under dry conditions due to the frictional mating contact inducing plastic deformation in relative motion, leading to a mating surface roughening and progressive wear, delamination, or even fatigue. In this regard, the friction of metallic materials is a crucial property to improve the performance, reliability, and efficiency of metallic materials contacts in various industries. Figure 9 shows the variation in friction coefficient of the specimens as a function of sliding distance. The friction coefficient of the specimens was increased drastically at the beginning of the test, but the friction coefficient of the LHT with UNSM treatment at HT specimen increased gradually. All of the specimens demonstrated running-in, transition, and steady-state periods, as partially shown each periods in Figure 9. Obviously, the untreated specimen had the highest friction coefficient among other specimens with a friction coefficient of 0.72 during running-in period, which reduced gradually till the friction coefficient of 0.58 in transition period, and then approached a stabilization in friction coefficient of 0.44 in the steady-state period. The friction coefficient of the UNSM-treated at RT specimen was also increased drastically at the beginning of the test, and then continued to increase to a friction coefficient of 0.62 during the running-in period and then reduced rapidly till the friction coefficient of 0.52 in transition period and then finally approached a stabilization in friction coefficient of 0.44 in steady-state period. The UNSM treatment was found to be beneficial in running-in and transition periods, but not in steady-state one, which is attributed to the lack of change in initial surface integrity of the UNSM-treated at RT specimen under dry conditions, where the effectiveness of UNSM treatment can be easily lost under severe plastic deformation. It has been reported earlier that the UNSM treatment reduced the friction coefficient of metallic materials under both oil-lubricated and dry conditions, due to the features of UNSM treatment, such as improvement in surface integrity, grain size refinement, the presence of micro-dimples on the surface, etc. [21,22,51]. On the contrary, Chen et al. have pointed out that refining the grain size of metallic materials cannot reduce the friction coefficient under dry conditions even though its` hardness may be increased significantly [52]. Fortunately, UNSM treatment not only increases the hardness of metallic materials, but it also reduces the surface roughness and creates a bunch of dimples (dint) that can behave as traps for wear particles under both oil-lubricated and dry conditions [51,54]. In case of the LHT without UNSM at HT specimen, the friction coefficient exhibited absolutely the same friction coefficient trend with the untreated and UNSM-treated at RT specimens, where the friction coefficient was about 0.51 in steady-state period, which was increased continuously throughout the friction test with relatively high fluctuation (see Figure 9). The continuous rise in friction coefficient is due to the generated wear particles or debris derived from roughened surface, due to repeated sliding under dry conditions. Interestingly, in the case of the LHT with UNSM at HT specimen, the friction coefficient slowly increased first to the highest friction coefficient of 0.59, and then reduced again to a value of 0.42, and finally approached stabilization in a friction coefficient of 0.39, which reduced slightly throughout the friction test. In addition, a shift in transition periods can be observed where the running-in and transition periods were found to be shortened by LHT with UNSM at HT, while a steady-state period achieved faster than other specimens, as shown in Figure 9. 

**Figure 10:**
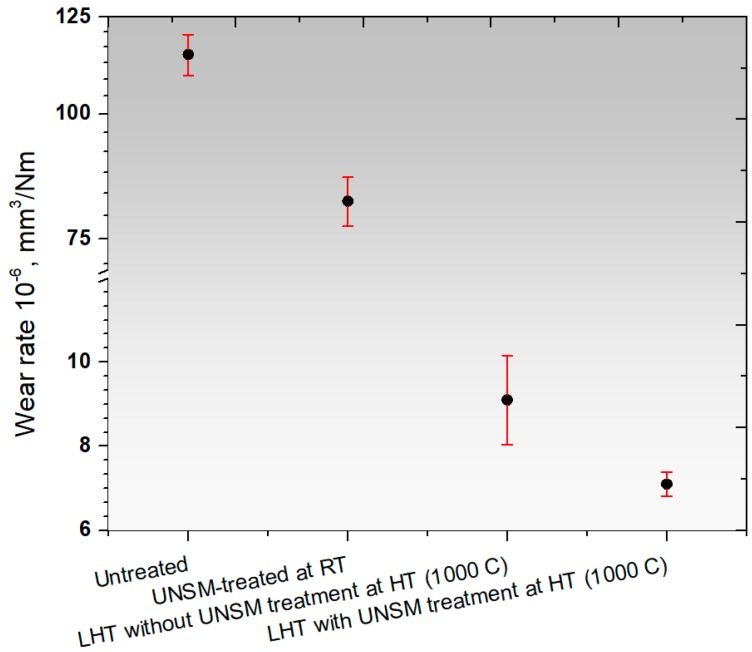
Comparison in wear rate of the untreated, UNSM-treated at RT and LHT without and with UNSM treatment at 1000 °C specimens.

The wear resistance of the specimens showing the rate how fast or slow wear occurred is shown in Figure 10, where the wear rate of the UNSM-treated at RT specimen was increased remarkably by about 18–20% in comparison with the untreated specimen. The corresponding wear resistance of the LHT without UNSM at HT specimen increased substantially in comparison with those specimens due to the increase in hardness, as shown in Figure 6. In turn, the corresponding wear resistance of the LHT with UNSM at HT specimen was further increased by an additional 36% in comparison with the LHT without UNSM at HT specimen. Overall, the corresponding wear rate of untreated specimen can be decreased by over 90% by the application of thermal-mechanical UNSM treatment at HT of 1000 °C. Wear resistance of a material and its hardness, compressive residual stress, and grain size have a linear correlation. The enhancement in wear resistance of the UNSM-treated specimen was attributed to the presence of gradient nanostructured surface layer, along with refined coarse grains into nano-sized grains, induced compressive residual stress, increased surface, and subsurface hardness. Furthermore, the wear resistance of the LHT with UNSM at HT specimen was further enhanced by increasing the UNSM treatment temperature up to 1000 °C in comparison with that of the UNSM-treated specimen. It was found that the combination of LHT with UNSM at HT of 1000 °C was able to produce a thicker gradient nanostructured surface layer, higher and deeper compressive residual stress, and also higher surface hardness and deeper subsurface hardness in comparison with that of the UNSM-treated specimen at RT. The development of LHT with UNSM treatment successfully demonstrated the possibility of further improvement in wear resistance of Ti-6Al-4V alloy by increasing the hardness, compressive residual stress, refining grain size in comparison with the UNSM-treated specimen at RT [26]. Moreover, it has been reported in our previous study that a gradient nanostructured surface layer with a thickness of about 60 µm was produced in Ti-6Al-4V by UNSM treatment at RT, while LHT with UNSM at HT of 800 °C was able to increase the thickness of nanostructured surface layer by up to about 100 µm [39]. 

**Figure 11:**
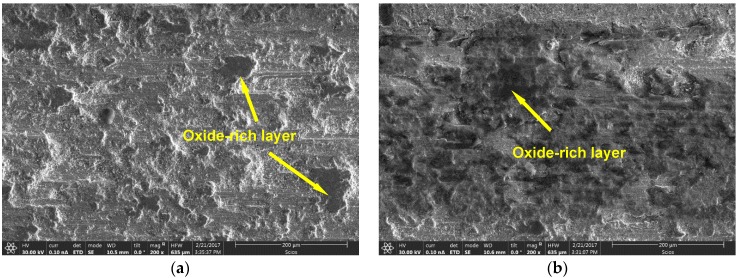
Scanning electron microscopy (SEM) images of the wear track generated on the surface of the UNSM-treated at RT (**a**) and LHT with UNSM treatment at HT of 1000 °C (**b**) specimens.

**Figure 12:**
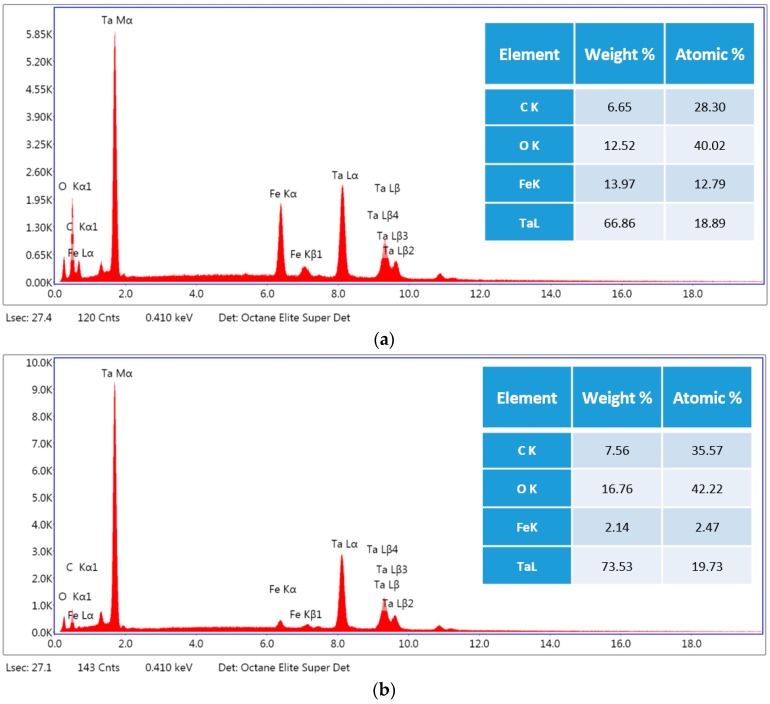
EDX (energy-dispersive X-ray spectroscopy) of the wear track generated on the surface of the UNSM-treated at RT (**a**) and LHT with UNSM treatment at HT of 1000 °C; (**b**) specimens.

The presence of gradient nanostructured surface layer was found to be responsible not only for the increase in wear resistance but also improvement in frictional behavior of the LHT with UNSM at HT specimen. In addition, in order to shed light on the friction and wear behavior, the surface morphology and the roughening after sliding distance of 30 m under dry conditions of the UNSM-treated at RT and LHT with UNSM at HT specimens are investigated as shown in Figure 11. It can be seen that the worn out deep scars and damages parallel to sliding direction were formed on the surface of the UNSM-treated at RT specimen that roughened the contact interface significantly without any cracks in comparison with the LHT with UNSM at HT specimen due to its high hardness. The average surface roughness inside the wear track of the UNSM-treated at RT and LHT with UNSM at HT specimens increased to 2.4 and 1.8 µm from its initial surface roughness of 0.08 µm. It means that the resistance against the sliding-induced surface roughening of the LHT with UNSM at HT specimen remained greater in comparison with the UNSM-treated at RT one, which is also owing to the presence of gradient nanostructured surface layer [54]. Moreover, the change in chemistry after wear test of the UNSM-treated at RT and LHT with UNSM at HT specimens is shown in Figure 12. It was found that the oxidative wear also played a crucial role in controlling the frictional behavior, where the amount of formed oxide-rich tribolayer was much more on the surface of the LHT with UNSM at HT specimen, as presented in the inset of Figure 12. Consequently, a newly developed thermo-mechanical UNSM treatment gives an opportunity to produce a gradient nanostructured surface layer with a thickness of several hundreds in microns, where the refined grain size is increased gradually at an incremental angle of about 30°, as shown in Figure 2d. Many researchers mentioned the advantages of gradient nanostructured surface layer over the nanostructured surface layer in terms of mechanical and thermal stabilities, and also strain localization [13,29,54].

## 4. Conclusions

In the current investigation, the effects of UNSM treatment with and without LHT at RT and HT (1000 °C) on the microstructure, hardness, and wear resistance of Ta were systematically investigated. The hardness of the UNSM-treated specimens at RT and HT (1000 °C) increased by about 20 and 62% in comparison with the untreated one. The LHT with UNSM at HT (1000 °C) was capable of inducing a greater compressive residual stress (~1400 MPa) at the surface layer in comparison with the UNSM-treated at RT and LHT without UNSM at HT. It was confirmed by cross-sectional EBSD observations that the combination of LHT with UNSM treatment at HT (1000 °C) produced a stable gradient nanostructured surface layer, with a thickness of several tens of microns, which led to an increase in wear resistance and a reduction in friction behavior of Ta. In general, the possibility of producing a stable gradient nanostructured layer by controlling the UNSM treatment temperature is significant and would find any potential applications of Ta in various industries. 

## Figures and Tables

**Table 1:** Mechanical and physical properties of Tantalum (Ta).

UTS, MPa	Yield Strength, MPa	Elastic Modulus, GPa	Poisson’s Ratio	Density, g/cm^3^	Elongation, %
900	170	186	0.35	16.6	5

**Table 2:** Ultrasonic nanocrystalline surface modification (UNSM) parameters at room temperature (RT) and high temperature (HT) (1000 °C).

Frequency, kHz	Amplitude, µm	Static Load, N	Speed, mm *ε*	Tip Material	Tip Diameter, mm	Interval, µm
20	40	30	2000	WC	2.38	70

**Table 3:** Wear test conditions under dry conditions.

Applied Normal Load, N	Reciprocating Speed, cm/s	Sliding Distance, m	Temperature, °C	Contact Stress, GPa
10	2.51	30	25	0.82
